# ABPI against Colour Duplex Scan: A Screening Tool for Detection of Peripheral Arterial Disease in Low Resource Setting Approach to Validation

**DOI:** 10.1155/2016/1390475

**Published:** 2016-03-10

**Authors:** Janaka Weragoda, Rohini Seneviratne, Manuj C. Weerasinghe, S. M. Wijeyaratne

**Affiliations:** ^1^Public Health Complex, Ministry of Health, 555/5, 6th Floor, Elvitigala Mawatha, Narahenpita, 10100 Colombo, Sri Lanka; ^2^Department of Community Medicine, Faculty of Medicine, University of Colombo, No. 25, Kynsey Road, 008000 Colombo, Sri Lanka; ^3^Department of Surgery, Faculty of Medicine, University of Colombo, No. 25, Kynsey Road, 00800 Colombo, Sri Lanka

## Abstract

*Background.* In Sri Lanka the ABPI has not been used as a screening tool to detect peripheral arterial disease (PAD) in epidemiological studies. This study was conducted to determine the best cutoff value of ABPI to detect PAD in Sri Lankan population.* Methods.* The ABPI measured by arterial Doppler to detect PAD was validated against colour duplex scan as the criterion using 165 individuals referred to vascular laboratory, National Hospital Sri Lanka. In all selected individuals ABPI was measured and lower limb colour duplex scan was performed. Narrowing of luminal diameter of lower limb arteries 50% or more was considered as haemodynamically significant and having PAD. The discriminative performance of the ABPI was assessed using Receiver Operator Characteristic (ROC) curve and calculating the area under the curve (AUC). The sensitivity and specificity of different threshold levels of ABPI and the best cutoff value of ABPI to detect PAD were determined.* Results.* ABPI 0.89 was determined as the best cutoff value to identify individuals with PAD. At this level of ABPI high sensitivity (87%), specificity (99.1%), positive predictive value (98.9%), and negative predictive value (88.4%) were observed.* Conclusion.* ABPI ≤ 0.89 could be used as the best cut off value to detect PAD.

## 1. Introduction

Peripheral arterial disease (PAD) is a slowly progressive atherosclerotic disease affecting vital organs of the body [1] and is usually characterized by occlusion of lower limb arteries ultimately causing acute or chronic limb ischemia [2]. PAD is the third most important atherosclerotic disease after coronary artery disease and cerebrovascular disease [3]. There are many traditional noninvasive tests available to assess PAD such as ABPI, Toe-Brachial Index, Segmental Pressure Examination, and pulse volume recordings. Colour duplex scan, magnetic resonance angiography, and computed tomographic angiography are new techniques available to diagnose PAD. However, these new methods are time consuming, expensive, and not easily accessible in low resource countries.

The ABPI is defined as the ratio of the highest ankle systolic blood pressure to highest brachial systolic blood pressure [4]. The ABPI is an easily applicable, less expensive, noninvasive test that can be used as the standard for the diagnosis of lower extremity PAD in field surveys, vascular laboratories, and clinic practice. The most important utility of the ABPI is at primary health care setting for the early detection and cost effective treatment of PAD [5]. Therefore, ABPI can be used for the screening tool for detecting PAD.

Although ABPI has been validated against lower extremity contrast angiography obtaining high sensitivity (85%–95%) and specificity (90%–100%), for diagnosing PAD [6–8] only a few studies describe the sensitivity and specificity of ABPI [8] at different levels. Literature is scarce on the use of ABPI as a screening tool to detect PAD in south Asian population. Further no studies were found on validation of ABPI among south Asian population.

The objective of this study was to assess the sensitivity and specificity of different threshold level of ABPI against diagnosis of PAD by colour duplex scan and to find out the best cutoff value of ABPI to detect PAD in Sri Lankan population.

## 2. Material and Methods

### 2.1. Study Design

This study was conducted in the vascular laboratory National Hospital, Sri Lanka, between September and December 2013. Those who are suspected of having PAD or other vascular disorders are referred to the vascular laboratory for conformation. The sample size was calculated using the formula for a descriptive study of a dichotomous variable based on the expected sensitivity and the specificity of the instrument, required level of precision, and the confidence level [9]. Calculated sample size was included 97 individuals with PAD and 68 individuals without PAD. From each selected individual only one lower limb was examined for the study. Thus, 165 individuals were selected from those who referred to the vascular laboratory. Ethics Review Committee, Faculty of Medicine, University of Colombo, granted the approval for the study. Informed consent was obtained from all selected individuals prior to participation.

The ABPI was validated for detection of PAD using colour duplex scan as the criterion. The colour duplex scan is a noninvasive technique for evaluation of anatomical location of the arterial occlusion and the degree of stenosis in lower extremities [10]. The quantitative criteria of the colour duplex scan used to diagnose severity of arterial stenosis were based on peak systolic velocity (PSV) and PSV ratios beyond the stenosis compared with the adjacent upstream segment. The PSV ratio from proximal to distal artery more than two (PSV distal/PSV proximal more than 2.0) was considered as a narrowing of luminal diameter of lower limb arteries of 50% or more and as having PAD. The PSV ratio more than 2 is commonly used to diagnose a stenosis greater than 50% diameter with sensitivity 95% to 100% [10]. Many other studies have reported high sensitive (95%–100%) and specificity (95%–100%) of colour duplex ultrasound scanning for detecting a significant arterial stenosis (>50% narrowing of luminal diameter) of lower limb arteries [11–13].

### 2.2. Data Collection

The arterial colour duplex scan of lower limbs was performed using Toshiba colour duplex ultrasound scan Model CC 15M71 to assess the degree of occlusion of lower limb arteries and to confirm PAD by a consultant vascular surgeon blinded to ABPI findings. Duplex scanning of lower limbs was carried out according to the procedure described by American society of echocardiography and the society of vascular medicine and biology [10]. Duplex ultrasound scan of lower limb arteries, common femoral artery, superficial femoral artery, popliteal artery, anterior tibial artery, and posterior tibial artery in both lower limbs were performed keeping the participant in supine position. The arterial spectral wave foam and peak systolic velocity (PSV) measurements were obtained from angle-corrected longitudinal spectral Doppler images using a curvilinear 7.5 MHz transducer. Images were acquired with the angle created by the direction of blood flow and the direction of the ultrasound beam kept at 60° or less. The spectral wave form and the PSV were recorded proximal to, at, and distal to the suspected stenosis. The PSV ratio from proximal to distal artery more than two (PSV distal/PSV proximal more than 2) was considered as a narrowing of luminal diameter of lower limb arteries of 50% or more and as having PAD [10]. Presence of calcified stenosis interferes with structural imaging of arteries in colour duplex scanning. But even in such instances visualization of a turbulent jet and velocity measurements were possible and thus detection of >50% diameter stenosis was enabled.

The sampling procedure was carried out till required number of individuals with and without PAD is selected for the study. Those who have undergone any surgery or procedure in lower limbs as a treatment of PAD were excluded from the study. The ABPI was measured in all selected individuals using Summit Vista ABI L 450VA Doppler instrument according to the procedure described by ACC/AHA guidelines for the management of patients with PAD [4]. The ABPI was calculated up to two decimals.

All selected individuals were interviewed with interviewer administered questionnaire (IAQ). Questions related to sociodemographic factors, general health condition, and smoking status were included in IAQ. Age was defined according to last birthday. Information on level of education, family income, smoking status, and questions related general health status such as diabetes mellitus, hypertension, dyslipidemia, coronary artery disease, and cerebrovascular disease was based on self-reporting. Claudication symptoms were assessed according to Edinburgh claudication questionnaire.

### 2.3. Statistical Analysis

The data analysis was performed by using the computer programme Statistical Package for Social Science (SPSS) version 16. The discriminative performance of the ABPI was assessed by Receiver Operator Characteristic (ROC) curve and by calculating the area under the curve (AUC). The ROC was drawn using ABPI as test variable and colour duplex scan as state variable. The optimal threshold level of ABPI which gives maximum correct classification of diseased and nondiseased was determined by the distance (*d* ^2^) between the point (0, 1) and any point on ROC curve. The *d* ^2^ was calculated for each observed threshold level of ABPI. The point of the minimum distance from the point (0, 1) to ROC curve was considered as the best cutoff level for ABPI to detect PAD.

## 3. Results

The median age of PAD group was 65 years (IQR: 59–68 years) and the median age of non-PAD group was 67 years (IQR: 62–70 years).  Table 1 shows more than 50% of those with PAD had history of diabetes mellitus, hypertension, or dyslipidemia which was significantly higher than those without PAD. A significantly higher proportion of current smokers (36.9%) were found among PAD than without PAD. Claudication symptoms were found to be only one-fifth of those with PAD.

**Figure 1:**
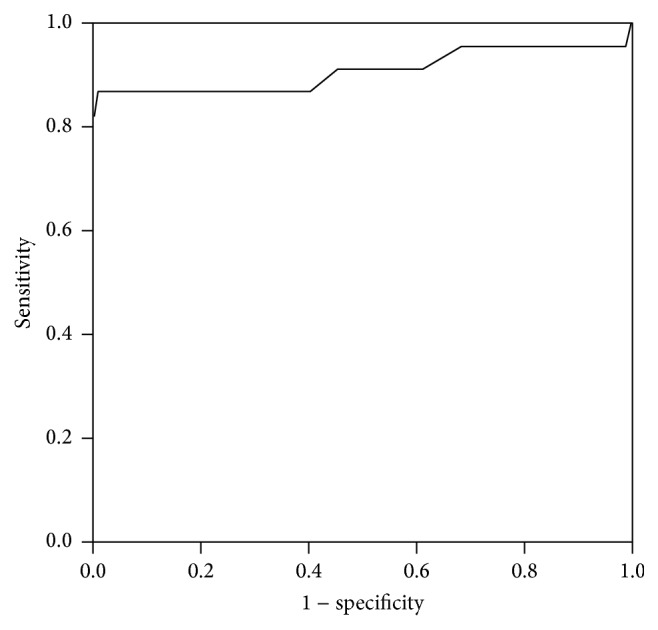
ROC curve for ABPI for having PAD among study population. Diagonal segments are produced by ties.

Figure 1 shows the ROC curve plotted for the different cutoff values of the ABPI giving the sensitivity against different values for 1 − specificity. The area under the curve (AUC) was 0.91 (95% CI: 0.81–1.01) as shown in Table 2. The AUC was significantly above and away from the diagonal reference line (*p* < 0.001) indicating the ability of the ABPI to differentiate those with and without PAD.

Table 3 shows the sensitivity and specificity at selected threshold levels of the ABPI according to the ROC curve. To obtain the optimal cutoff value to discriminate disease with nondisease subject, distance from curve (*d* ^2^) was calculated for each observed threshold level of ABPI. The *d* ^2^ for different threshold levels of ABPI is shown in the 4th column of the Table 3. When the threshold level of ABPI is increased for detecting PAD, the sensitivity increases and specificity decreases. At the level of 0.85 the specificity of detecting PAD was 100% and sensitivity was 82.6%. At the level of ABPI equal or more than 1.18 (ABPI ≥ 1.18) the sensitivity of detecting PAD was above 95% and specificity below 32%. The shortest *d* ^2^ between the point (0, 1) and any point on ROC curve was 0.0169 where maximum AUC of the ROC is observed. The corresponding ABPI at maximum AUC was 0.89. Therefore, the best cutoff value of ABPI to identify individuals with PAD was 0.89.

Table 4 shows the sensitivity, specificity, positive predictive value, and negative predictive value which are validity indicators of the ABPI at 0.89 cutoff level. Having a sensitivity of 87.0% and a specificity of 99.1% was indicative of good detection ability of the ABPI.

## 4. Discussion

The objective of this study was to assess the sensitivity and specificity of different threshold levels of ABPI in detecting PAD and to find out the best cutoff value of ABPI to detect PAD in Sri Lankan population. Many studies have reported more than 95% of sensitivity and specificity of colour duplex ultrasound scan in detecting a significant stenosis of the arteries [10–13] and it is the most commonly used method to diagnose PAD in Sri Lanka. Thus, we decided to validate ABPI against colour duplex scan for detecting PAD.

In many studies [14–17] ABPI ≤ 0.9 has been used as the criterion for diagnosing PAD based on ACC/AHA guidelines for the management of patients with PAD [4]. However, there is a borderline category where ABPI ranges from 0.91 to 0.99, a range which does not clearly categorize an individual having or not having PAD. Although traditionally the cutoff value of ABPI for defining the presence of PAD is ≤0.90, those with ABPI values between 0.90 and 1.10 may have early or mild lower extremity atherosclerosis [18]. In addition, at the cutoff value of ABPI ≤0.9 it has been reported that the sensitivity of diagnosing PAD was 83% to 85% and a specificity of 95% to 100% [6, 8, 18, 19].

This study found that at the level of ABPI ≤ 0.85 the sensitivity of detecting PAD was 82.6% and specificity was 100%. We observed the ABPI ≤ 0.89 as the best cutoff value to identify those with PAD which is very close to the ABPI ≤0.9, the cutoff value recommended by ACC/AHA guidelines to diagnose PAD [4]. Further, sensitivity (87%) and specificity (99.1%) of detecting PAD at ABPI 0.89 were compatible with many other studies [6, 8, 18, 19]. In the range of ABPI 1.18–1.28 the sensitivity of diagnosing PAD was more than 95% with a low specificity ranging from zero to 31%. Similar to these findings Lijmer et al. [8] have reported at the level of ABPI 1.19 the sensitivity of 94% and 29% specificity for detecting PAD. Hence, we found at the ABPI ≥ 1.18 the best cutoff level to exclude PAD.

## 5. Conclusion

This study provides evidence to use ABPI as a valid method to screen PAD with high level of sensitivity and specificity in low resource primary and secondary health care settings.

## Figures and Tables

**Table 1:** Participant characteristics by presence of PAD.

Character	PAD (*n* = 97)		Non-PAD (*n* = 68)		*p* value
	No	%	No	%	
Age					
45–54	17	17.5	05	7.4	0.162
55–64	32	33.0	24	35.3	
65–74	48	49.5	39	57.3	
Sex					
Male	46	47.4	41	60.3	0.103
Females	51	52.6	27	39.7	
Highest educational level					
≤Grade 5	16	16.5	12	17.6	0.824
Grade 6–10	27	27.8	16	23.5	
GCE O/L completed and above	54	55.7	40	58.8	
Monthly family income Rs					
<30,000	32	33.0	33	48.5	0.129
30,000–50,000	32	33.0	18	26.5	
>50,000	33	34.0	17	25.0	
Diabetes mellitus (yes)	49	50.5	08	11.8	<0.001
Hypertension (yes)	53	54.6	18	26.5	0.001
Dyslipidemia (yes)	54	55.7	15	22.1	<0.001
Coronary artery disease (yes)	08	8.2	03	4.4	0.115
Cerebrovascular accidents (yes)	09	9.3	03	4.4	0.336
Claudication symptoms (yes)	19	19.6	02	2.9	<0.001
Smoking status among male participants					
Current smokers	17	36.9	05	12.1	0.014
Former smokers	15	32.6	18	43.9	0.890

**Table 2:** Statistics of the area under the curve of the ROC curve for the ABPI against diagnosis of PAD by arterial colour duplex scan.

AUC	Standard error^a^	Asymptomatic Sig.^b^	Asymptomatic 95% CI	
			Lower bound	Upper bound
0.91	0.051	0.000	0.809	1.01

^a^Under the nonparametric assumption. ^b^Null hypothesis: true area = 0.5.

**Table 3:** Sensitivity and specificity for selected threshold levels of levels of ABPI for detecting a ≥ 50% narrowing of arterial luminal diameter anywhere in the lower limb.

Threshold levels (ABPI)	Sensitivity	Specificity	Distance from the curve (*d* ^2^)
0.83	73.9	100	0.0681
0.85	82.6	100	0.0302
0.86	82.6	99.5	0.0303
0.89	87.0	99.1	0.0169
0.91	87.0	98.6	0.0170
0.93	87.0	98.2	0.0172
0.94	87.0	97.7	0.0174
1.17	91.3	39.2	0.3772
1.18	95.7	31.8	0.4669
1.25	95.7	4.1	0.3499
1.26	95.7	1.4	0.7414
1.27	1.000	0.5	0.9900
1.28	1.000	0	1.0000

ABPI: ankle brachial pressure index.

**Table 4:** Validity of the ABPI of 0.89 as an indicator of PAD.

Cutoff value	Sensitivity	Specificity	PPV	NPV	LR+	LR−
0.89	87.0%	99.1%	98.9%	88.4%	96.6	0.13

PPV: positive predictive value, NPV: negative predictive value, LR+: likelihood ratio positive, and LR −: likelihood ratio negative.
